# Enhanced detergent extraction for analysis of membrane proteomes by two-dimensional gel electrophoresis

**DOI:** 10.1186/1477-5956-3-5

**Published:** 2005-06-07

**Authors:** Matthew A Churchward, R Hussain Butt, John C Lang, Kimberly K Hsu, Jens R Coorssen

**Affiliations:** 1 Dept. of Physiology and Biophysics, University of Calgary Faculty of Medicine, 3330 Hospital Drive NW, Calgary AB, T2N 4N1, CANADA; 2 Dept. of Biochemistry and Molecular Biology, University of Calgary Faculty of Medicine, 3330 Hospital Drive NW, Calgary AB, T2N 4N1, CANADA; 3 Hotchkiss Brain Institute, University of Calgary Faculty of Medicine, 3330 Hospital Drive NW, Calgary AB, T2N 4N1, CANADA

## Abstract

**Background:**

The analysis of hydrophobic membrane proteins by two-dimensional gel electrophoresis has long been hampered by the concept of inherent difficulty due to solubility issues. We have optimized extraction protocols by varying the detergent composition of the solubilization buffer with a variety of commercially available non-ionic and zwitterionic detergents and detergent-like phospholipids.

**Results:**

After initial analyses by one-dimensional SDS-PAGE, quantitative two-dimensional analyses of human erythrocyte membranes, mouse liver membranes, and mouse brain membranes, extracted with buffers that included the zwitterionic detergent MEGA 10 (decanoyl-N-methylglucamide) and the zwitterionic lipid LPC (1-lauroyl lysophosphatidylcholine), showed selective improvement over extraction with the common 2-DE detergent CHAPS (3 [(3-cholamidopropyl)dimethylammonio]-1-propanesulfonate). Mixtures of the three detergents showed additive improvements in spot number, density, and resolution. Substantial improvements in the analysis of a brain membrane proteome were observed.

**Conclusion:**

This study demonstrates that an optimized detergent mix, coupled with rigorous sample handling and electrophoretic protocols, enables simple and effective analysis of membrane proteomes using two-dimensional electrophoresis.

## Background

Historically, the proteomic analysis of hydrophobic membrane proteins has been considered to be difficult within the bounds of conventional protocols for two-dimensional gel electrophoresis (2-DE). The nature of first dimension isoelectric focusing (IEF) requires that proteins be thoroughly solubilized as they are subjected to an electric field in which they migrate to their isoelectric point, by definition the state of lowest possible net charge and thus lowest solubility in aqueous environments. In addition to being highly hydrophobic, many integral membrane proteins tend to be very large: human Ca^2+ ^channels have 24 transmembrane helices and are typically > 200 kDa [1], and tyrosine kinase receptors are frequently > 100 kDa [2,3]. This leads to two major problems in the preparation of membrane protein samples for 2-DE. First, effectively extracting membrane proteins into a detergent that is IEF compatible. Second, maintaining protein solubility throughout loading onto IPG strips and the subsequent first dimension IEF separation. Although highly efficient membrane protein extractions are routinely carried out with a detergent such as SDS for one-dimensional PAGE, SDS is incompatible with IEF due to the charged head group. To overcome this, SDS solubilized samples often undergo solvent or acid precipitation to remove or reduce SDS and lipids. Despite these harsh treatments and even subsequent treatment of the precipitate with a strong base [4], delipidation by solvent extraction is often cited as enhancing protein recovery [5,6] without discussion of the loss or modification of proteins during precipitation. For example, highly hydrophobic proteins (such as proteolipids) and proteins with particular post-translational modifications (such as palmitoylation) are capable of partitioning into the solvent phase [7-9], and TCA treatment can cause acid hydrolysis of proteins or alter post-translational modifications. Additionally, some early general problems with effectively separating hydrophobic proteins by 2-DE have led to widespread general disregard for the analysis of membrane proteins, particularly in the development of alternate proteomic approaches [4,5,10,11].

Since membrane proteins comprise approximately 30% of human proteins[12], and may account for substantially more cellular functions, the focus on soluble proteins in so-called 'full' proteomic analyses is somewhat concerning. There is evidence that optimization of extraction conditions by alteration of buffers, chaotropes, and detergents is sufficient to reliably achieve high-resolution maps of membrane proteins [13-16]. To this end we have sought simple alternatives to optimize the detergent conditions used to extract proteins from native membranes by systematic analysis of the solubilization properties of a wide range of commercially available non-ionic and zwitterionic detergents and a range of natural and synthetic detergent-like lipids [17-19]. Using proven synthetic detergents, together with more native lipophilic agents, we find that combinations of these reagents generally improve the resolution of membrane proteomes analyzed by 2-DE, providing for select improvements in the yields of specific proteins. Optimization of conditions for particular samples remains a key to any successful analysis [20-22].

## Results & Discussion

### 1D SDS-PAGE of RBC membrane

**Figure 1:**
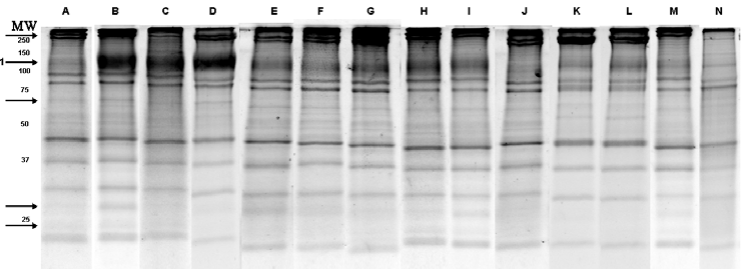
Composite of 1D SDS-PAGE analyses of RBC ghost membranes extracted with A) 4% CHAPS, B) 2% SDS, C) 2% LPC, D) 2% lauric acid, E) 2% trans, trans-farnesol, F) 2% MEGA 8, G) 2% MEGA 9, H) 2% MEGA 10, I) 2% 1,2 dioleoyloxy -3-(dimethylamino)propane, J) 2% SB 3–10 (Sigma), K) 2% SB 3–10 (Calbiochem), L) C12E8, M)1-oleoyl-sn-glycerol, N) DL-α-O-benzylglycerol. Arrows indicate notable differences between extractions including 1, the multiple transmembrane spanning protein band III.

Analysis of RBC extracts using 1D SDS-PAGE allowed for the rapid screening of a large number of extraction reagents (including glycerols, lipids, fatty acids, and isoprenoids), providing results that could be interpreted qualitatively based on the selective increase and decrease of protein banding patterns relative to control extractions with CHAPS or SDS (Fig. 1). For example, band III, a large protein with multiple transmembrane spanning domains [23] could be clearly distinguished in SDS extracts at an apparent MW of ~110 kDa, compared to those made with CHAPS. Another band, at apparent MW of 28 kDa, was also observed in the SDS but not the CHAPS extract. Based on these simple criteria, detergents were selected that gave improved banding patterns over the CHAPS control extraction. An initial working series of effective detergents was thus identified for further testing (Table 1), and these were then used to extract RBC membrane samples for subsequent analysis by 2-DE. Notably LPC, the N-methylglucamide detergents MEGA 8, 9, and 10 and the sulfobetaine-based detergents ASB-14 and SB 3–10 showed improvements in the 1D banding pattern relative to the CHAPS control. A selection of natural source lipids were also tested, including lysophsphatidylglycerol (LPG), lysophosphatidylethanolamine (LPE), lysophosphatidylcholine (LPC, from egg), lysophosphatidylserine (LPS, from bovine brain), and cardiolipin (bovine heart). Although generally comparable, and of some selective use in extractions, most of these natural lipids proved to be of limited general usefulness as they are charged at neutral pH, and thus inherently incompatible with IEF. This limitation does not obviate the potential application of these lipids as extraction agents for use with alternate protein separation paradigms.

**Table 1:** Summary of detergents tested using systematic 1D SDS-PAGE analysis. Overall extraction efficacy analyzed by 1D SDS-PAGE or 2-DE separation is expressed qualitatively relative to SDS extraction (for 1D analysis) or CHAPS extraction (for 2-DE). + indicates compatibility but poor perfomance, ++ indicates similar or slightly worse than CHAPS extraction, +++ indicates performance equal to or better than CHAPS, – indicates incompatibility.

Detergent	1D-PAGE	2-DE	Comments & Rationale
SDS	+++	-	IEF incompatible
CHAPS	++	+++	Poor extraction of hydrophobic and high molecular weight proteins
*trans, trans*-farnesol	+++	++	Natural isoprenoid
MEGA-8^a^	+++	++	Group of nonionic detergents commonly used for protein purification [35,36]
MEGA-9^b^	+++	++	
MEGA-10^c^	+++	+++	
amidosulfobetaine-14 (ASB-14)	+++	++	Sulfobetaine-based detergents reported to improve membrane protein extraction [24-26]
Zwittergent^® ^3–10/SB 3–10^d^	+++	+++	
LPC (synthetic, lauroyl chain)^e^	+++	+++	Zwitterionic lysophospholipid
LPC (egg, mixed chain)^e^	++	++	Zwitterionic lysophospholipid
LPS (bovine brain)^f^	++	-	Anionic lysophospholipid, incompatible with IEF
LPE (egg, mixed chain)^g^	-	-	Zwitterionic lysophospholipid; low solubility in high urea buffer
LPG (egg, mixed chain)^h^	++	-	Anionic lysophospholipid, incompatible with IEF
LPA (egg, mixed chain)^i^	++	-	Anionic lysophospholipid, incompatible with IEF
cardiolipin (bovine heart)	++	-	Anionic lipid, incompatible with IEF, low solubility in high urea buffer
5,7-docosadiynoic acid	-	-	Synthetic fatty acid; low solubility in high urea buffer
lauric acid	+++	++	Medium chain fatty acid; low solubility in high urea buffer
free fatty acids (mixed)	-	-	Mixed natural fatty acids; low solubility in high urea buffer
DODAP^j^	+++	-	Cationic lipid used as a transfection reagent [37], IEF incompatible
1-oleoyl-*sn*-glycerol	+	-	Uncharged monoacylated lipid, very low solubility in high urea buffer
C_12_E_8_^k^	+	-	Nonionic detergent used to study membrane proteins [38]
DL-α-O-benzylglycerol	+	-	Amphipathic cyclic glycerol conjugate
tryptophol	++	-	Amphipathic heterocyclic metabolite of tryptophan; ionizable at low pH

^a ^octanoyl-N-methylglucamide

^b ^nonanoyl-N-methylglucamide

^c ^decanoyl-N-methylglucamide

^d ^N-decyl-N,N-dimethyl-3-ammonio-1-propanesulfonate

^e ^L-α-lysophosphatidylcholine

^f ^L-α-lysophosphatiylserine

^g ^L-α-lysophosphatidylethanolamine

^h ^L-α-lysophosphatidylglycerol

^i ^L-α-lysophosphatidic acid

^j ^1,2-dioleoyloxy-3-(dimethylamino)propane

^k ^octaethylene glycol monododecyl ether

### 2-DE analysis of Red Blood Cell membrane

**Figure 2:**
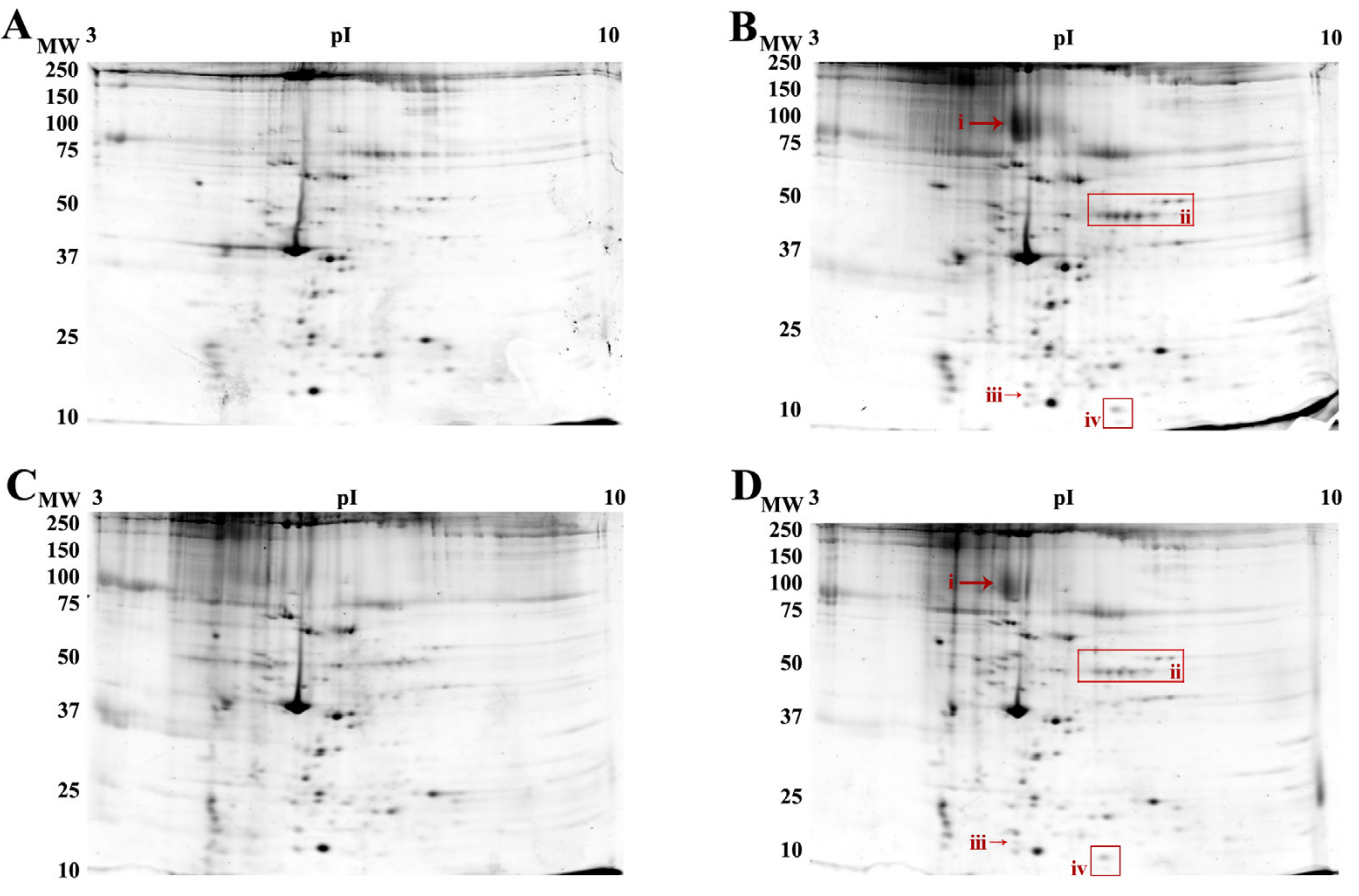
2-DE of RBC ghost membranes extracted with A) 4% CHAPS, B) 3% CHAPS : 1% LPC, C) 3% CHAPS : 1% MEGA 10, D) 3% CHAPS : 0.5% LPC : 0.5% MEGA 10. Extractions were carried out in buffer with 8 M urea, 2 M thiourea, protease inhibitor cocktail, and the indicated detergent for 1 hour on ice. Gels are representative of three independent experiments. Roman numerals indicate areas of improvement including i, the multiple transmembrane spanning protein band III.

**Figure 3:**
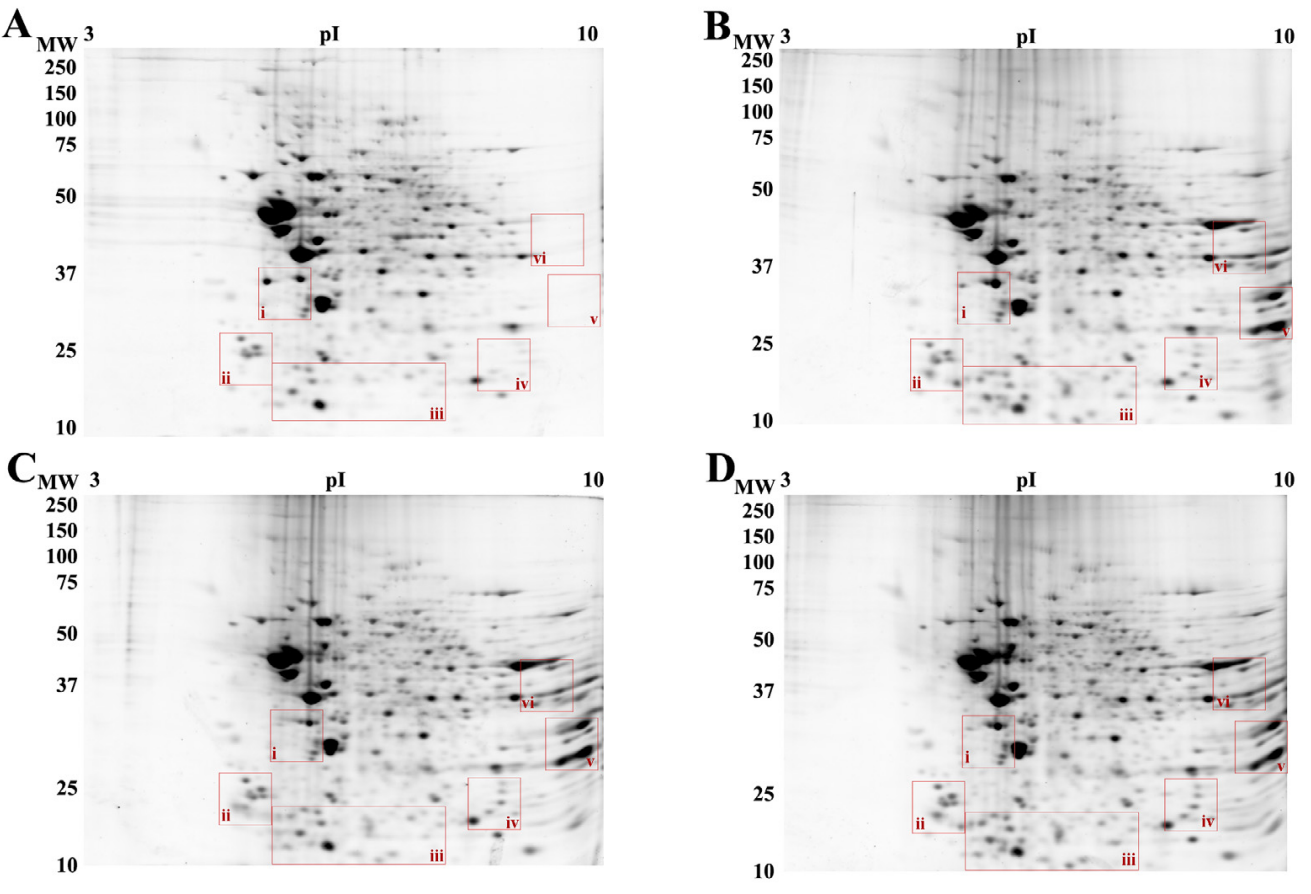
2-DE of mouse brain membranes extracted with A) 4% CHAPS, B) 3% CHAPS : 1% LPC, C) 3% CHAPS : 1% MEGA 10, D) 3% CHAPS : 0.5% LPC : 0.5% MEGA 10. Extractions were carried out as for Fig. 2. Gels are representative of three independent experiments. Areas defined with Roman numerals are shown in Fig. 4.

**Figure 5:**
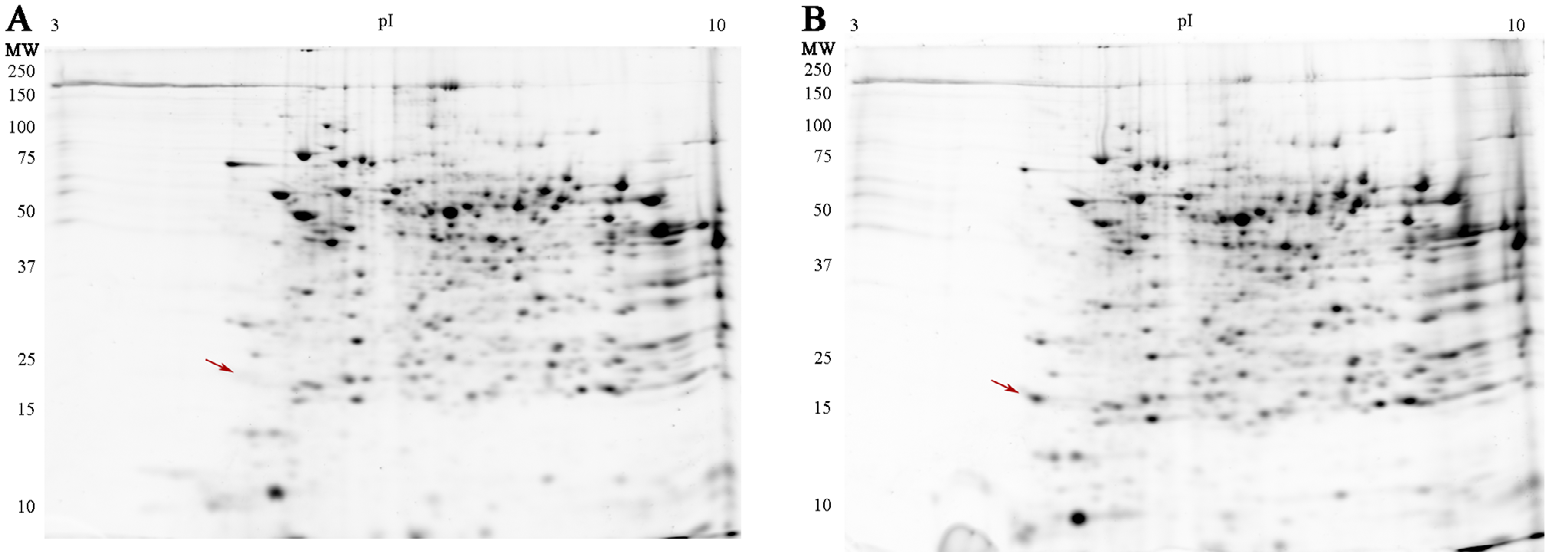
2-DE gels of mouse liver membranes extracted with A) 4% CHAPS, B) 3% CHAPS : 1% LPC. Extractions were carried out as for Fig. 2. Gels are representative of three independent experiments. Arrow indicates specific differences between gels.

Initially, red blood cell (RBC) membranes were extracted with a 2-DE buffer containing 4% total CHAPS (our standard concentration) or 1–2% total detergent, due to the relatively lower solubility of most test detergents compared to the highly soluble CHAPS. Extraction of RBC membranes with synthetic LPC (lauroyl chain, Sigma) and the zwitterionic detergent MEGA 10 under these conditions resulted in areas of selective improvement in resulting 2-DE patterns relative to the control extracts in CHAPS (data not shown). 2-DE of samples extracted with SB 3–10 yielded similar protein maps to CHAPS, however this detergent was difficult to solubilize into high urea buffer, as previously reported [24,25]. However, contrary to earlier reports [24,26], samples extracted with ASB-14 did not show improvement over either CHAPS or SB 3–10 (data not shown). In order to both appropriately account for more general effects of detergent concentration and take advantage of the high solubility and efficient solubilizing properties of CHAPS, both LPC and MEGA 10 were used to extract RBC membranes (Fig. 2), mouse brain membranes (Fig. 3), and mouse liver membranes (Fig. 5) as mixtures of 3% CHAPS : 1% alternate detergent, for a total 4% detergent.

RBC membranes extracted with the 4% total detergent mixtures of CHAPS and LPC showed general evidence of improved spot densities (Fig. 2B), as well as specific improvements in terms of reduced horizontal streaking in the intermediate molecular weight region (Fig. 2A). Automated spot detection and quantitative comparative analysis using Progenesis Workstation software identified specific changes in the protein pattern. Specific areas of the gels showed improvement relative to parallel 4% CHAPS control gels (Fig. 2Bi–iv). In particular, a prominent spot corresponding to band III was clearly observed [15,16], as well as a 2.2 ± 0.1 – fold increase in the density of a string of spots relative to the CHAPS extract (Fig. 2Bii). Three additional unique spots were observed when extracted with 3% CHAPS : 1% LPC (Fig 2Biii–iv). RBC membrane samples extracted with 3% CHAPS : 1% MEGA 10 (Fig. 2C) yielded protein maps of generally equivalent resolution to both the 3% CHAPS : 1% LPC and the 4% CHAPS maps, but did not resolve the protein band III as effectively as 3% CHAPS : 1% LPC.

In order to examine the overlapping effects of both these test detergents, but ameliorate the observed losses of protein, 0.5% of each was mixed with 3% CHAPS and tested in the extraction and 2-DE analysis of RBC membrane proteins (Fig. 2D). Extraction with 3% CHAPS : 0.5% LPC : 0.5% MEGA 10 does not yield the extent of differences identified in the 3% CHAPS : 1% LPC extracted condition, although the maps show general improvements over the control CHAPS condition that correlate with the improvements seen in the two individual detergent extractions (Figs. 2B, C). The density of the indicated string of proteins was increased an average of 1.7 ± 0.2 – fold over CHAPS (Fig. 2Dii). In general then, the addition of LPC to the extraction buffer enhances both protein recovery and resolution in the subsequent 2D protein maps.

Our initial findings using the RBC membrane as a model system lead us to expand the analyses to additional tissue types. Mouse brain membranes [27] and mouse liver membranes were chosen due to their availability, and broad international interest in improved analyses of these tissue proteomes.

### 2-DE analysis of mouse brain membrane

**Figure 4:**
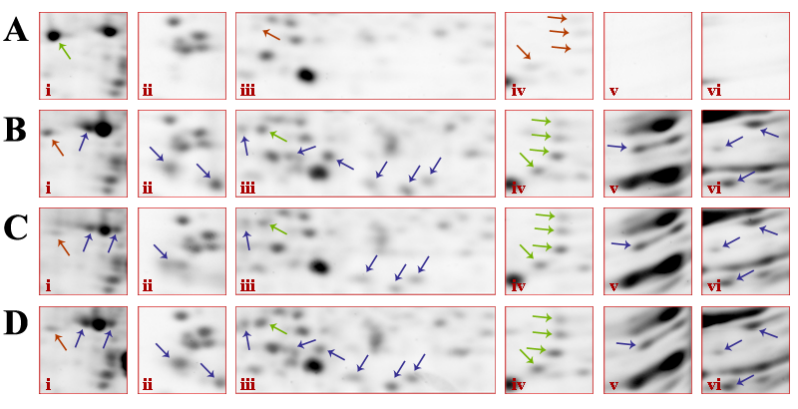
Enlargement and contrast of selected regions after 2-DE of mouse brain membranes (see areas defined in Fig 3). Areas i-vi show selective increases in spot number, resolution, and density. Samples were extracted with A) 4% CHAPS, B) 3% CHAPS : 1% LPC, C) 3% CHAPS : 1% MEGA 10, D) 3% CHAPS : 0.5% LPC : 0.5% MEGA 10. Results are representative of three independent experiments. Green arrows indicate spots showing increased volume and density, red arrows indicate decrease, blue arrows indicate novel spots.

Adult mouse brain membrane samples were subjected to the final four extraction conditions (Fig. 3) in order to further test the results obtained in RBC membranes (Fig. 2). Overall the results were quite similar to those obtained in the tests on RBC membranes. Extraction of mouse brain membranes with 3% CHAPS : 1% LPC (Fig. 3B) showed improvement of spot number, density and resolution compared to extraction with 4% CHAPS alone (Fig. 3A); quantitative analysis indicated specific areas of significant improvement (Fig. 4). Automated analysis identified 13 ± 3 novel spots that were reproducibly detected primarily in the low molecular weight and basic extreme regions of the gel (Fig. 4B; blue arrows indicate novel spots). Additionally, 5 spots were identified that significantly increased in volume an average 7.0 ± 3.4 -fold, and increased in density 2.8 ± 0.9-fold compared to the 4% CHAPS condition (Fig. 4B; green arrows indicate increased recovery). Of the 15 ± 2 novel spots detected in the 3% CHAPS : 1% MEGA 10 condition (Fig. 4C), most were also observed in the 3% CHAPS : 1% LPC condition. Overall, of the same 5 spots showing increased recovery, the volume increased 5.8 ± 2.5-fold, while density increased 3.2 ± 0.9-fold (Fig 4C; green arrows). Extraction of mouse brain membrane with 3% CHAPS : 0.5% LPC : 0.5% MEGA 10 (Fig. 3D) showed an additive effect on spot number. Spots recovered in both 3% CHAPS : 1% LPC and 3% CHAPS : 1% MEGA 10 were also detected in the combined extraction system. 13 ± 1 novel spots were detected relative to control, and the 5 previously identified spots increased in volume 6.4 ± 0.4-fold and density was increased 2.6 ± 0.6-fold (Fig 4D; green arrows). The nature of the recovery of these protein spots in 3% CHAPS : 0.5% LPC : 0.5% MEGA 10 reveals the specific action of the two detergents – LPC and MEGA 10 working in concert. Only one selective loss of a protein spot was observed in relation to this recovery of unique spots (Fig. 4Ai); this loss is the result of a specific action shared by the two alternate detergents as opposed to a result of the difference in CHAPS concentration during extraction since this protein was not recovered even after extraction with 5% total detergent (4% CHAPS : 0.5% LPC : 0.5% MEGA 10) (data not shown). This loss implies some specific action of the alternate detergents that prevent the extraction of this particular protein, or possibly an alteration in the electrophoretic mobility of this protein in the first dimension by means of increasing or decreasing the number of exposed ionizable residues. Together, the results of the RBC membrane and mouse brain membrane extractions show that simple combinations of zwitterionic detergents (CHAPS and MEGA 10) with a zwitterionic lipid (LPC) are generally more effective at extracting membrane proteins and maintaining protein solubility during first dimension IEF than are standard CHAPS-based extraction conditions.

### Additional 2-DE Analyses

Interestingly, extracting mouse liver membranes with the same detergent combinations described above resulted in protein maps that were highly similar, with very limited improvements. Automated analysis indicated almost complete overlap of the resulting 2-DE protein patterns (Fig 5), with the specific and substantial recovery of one additional protein spot. We interpret the marked similarity in these liver protein profiles, relative to the differences seen in the RBC and brain samples, to be due to variability between tissues in terms of relative homogenization/extraction efficiency and compatibility with our current buffer system.

To control for possible differences arising from the changing CHAPS concentration in these test extraction buffers, mouse brain membranes were also extracted with 5% total detergent (5% CHAPS or 4% CHAPS : 0.5% LPC : 0.5% MEGA 10) and analyzed in parallel with membranes extracted with 4% total detergent. No significant difference in overall spot pattern or specific differences as described above was observed between the 5% and the 4% total detergent mixtures (data not shown), indicating that the differences described here are specifically attributable to the addition of LPC and MEGA 10 as solubilizing agents. Indeed, overall, membrane protein patterns were generally of somewhat lower resolution when the CHAPS concentration or total detergent concentration was increased to 5%.

### Protein Quantification

During initial experiments we found total protein load to be the most significant variable confounding quantitative analyses. As such, great care was taken to ensure that the analyses meaningfully tested protein extraction and solubilization efficiency, in isolation from complicating variables. Simply, the goal was to compare reagents and conditions, not to compare different final total protein loads by 2-DE. Initially many protein samples were quantified using a modified Folin total protein assay (RC DC Protein Assay kit, BioRad). Colourimetric assays of this type (eg. Bradford, Lowry, BCA, and so forth) perform acceptably under many circumstances requiring routine normalization of a series of very similar samples. However one of several limitations of such total protein assays is a marked sensitivity to interfering substances, including components of typical IEF solubilization solutions such as detergents, reducing agents, and urea. In our experiments, detergents and detergent concentrations were systematically altered and combined. Not unexpectedly, we observed substantial variability in the results of the total protein assay, depending upon the solubilizing reagents present. The complications of applying systematic corrective controls, or of preparing separate standard curves for each of the solubilization conditions tested, simply increased the potential for error. Regardless, separate standard curves are not even feasible in the case of the RC DC assay, as urea causes a saturating false positive signal.

We have found that the EZQ Protein Quantitation kit (Molecular Probes) is insensitive to the nature and concentrations of detergent in all samples tested. In this assay format, the immobilized protein sample is washed exhaustively with methanol to remove components of the solubilization solution prior to addition of the fluorescent protein detection reagent. Thus, the chemistry of the assay proceeds in the absence of potentially confounding contaminants. In extensive comparisons, there were no significant differences in standard protein assay curves regardless of the type or quantity of detergent included (data not shown). Additionally, the method proved quite sensitive (routine detection of 0.030 μg of total protein/spot, or 15 μg/ml); this is fully 10-fold more sensitive and requires 4-fold less material than the RC DC Assay. Thus, as the chemistry of the assay was not altered under our different experimental conditions, we are confident that the improvements observed in our final protein maps were truly the result of differences in extraction and solubilization efficiency, and not artifacts generated by erroneous total protein assays leading to inconsistent total protein IEF loads between different test conditions. Although the EZQ protein assay certainly has its caveats, not least of which is cost, it does offer distinct benefits that support its utility in these and other ongoing proteomic analyses.

## Conclusion

In order to optimize recovery of hydrophobic proteins for 2-DE, we have sought a simple, direct solution to the problem of protein extraction and solubility during IEF. The systematic screening and combination of commercially available detergents offers a direct, inexpensive, and convenient method for optimizing the conditions of IEF without entering into the complexities of a systematic synthesis of new detergents based on specific base molecules, or the potential losses or modification of proteins associated with solvent extraction techniques. Coupled with our ability to effectively analyze membrane proteomes using 2-DE [27] the resulting findings should also prove of use in defining optimized combinations of extraction reagents for use with alternate protein separation protocols.

Based on the hypothesis that highly lipophilic molecules (albeit at lower total concentrations than can be achieved with the more standard detergents), might better mimic native lipid-membrane protein interactions and thus improve protein solubilization, we found that LPC can substantially augment the extraction of membrane proteins from different sources. This finding does not obviate the need for optimization of extraction and 2-DE conditions for different samples, but does provide a powerful, widely available and reasonably priced alternative that can be readily tested in parallel with more routine solubilization reagents. Rigorous testing of protein assays ensured that these findings reflect a true effect on extraction and protein solubility, rather than an artifact of inconsistent protein loads between different 2-DE analyses. Notably, LPC and MEGA 10 provided particularly marked improvements in the resolution of the mouse brain membrane proteome.

## Methods

### Reagents

L-α-lysophosphatidylcholine lauroyl, urea, tris acetate, lauric acid, pH 3–10 ampholytes, ammonium persulfate, decyl-N,N-dimethyl-3-ammonio-1-propanesulfonate (SB 3–10), amidosulfobetaine-14 (ASB-14), DL-α-O-benzylglycerol, tributylphosphine (TBP), HEPES, sodium orthovanadate, staurosporine, cantharidin, and components of the broad spectrum protease inhibitor cocktail [28] were purchased from Sigma (St. Louis, Missouri). IPG strips (pH 3–10), 30% acrylamide/bisacrylamide solution, low melting agarose, Sypro Ruby, 10×TGS running buffer, RC DC Protein Assay Kit, bovine γ-globulin, and SDS were from BioRad (Hercules, California). EZQ Protein Quantitation Kit was from Molecular Probes (Eugene, OR), Zwittergent^® ^3–10 was from Calbiochem (La Jolla, California), and CHAPS was from Anatrace (Maumee, Ohio). 1,2-dioleoyloxy-3-(dimethylamino)propane, 5,7-docosadiynoic acid, and 1-oleoyl-*sn*-glycerol were from Toronto Research Chemicals (Toronto, Ontario). Bovine brain L-α-lysophosphatidylserine, egg L-α-lysophosphatidylcholine, egg L-α-lysophosphatidylethanolamine, egg L-α-lysophosphatidylglycerol, egg L-α-lysophosphatidic acid, bovine heart cardiolipin, and free fatty acids were from Doosan Serdary Research (Toronto, Ontario). Thiourea was from Fisher Scientific (Hampton, New Hampshire), and PBS, DTT, octanoyl-N-methylglucamide (MEGA 8), nonanoyl-N-methylglucamide (MEGA 9), decanoyl-N-methylglucamide (MEGA 10), TEMED, glycerol, 40% acrylamide solution, and octaethylene glycol monododecyl ether (C_12_E_8_) were from Bio Basic Inc. (Markham, Ontario). Narrow range ampholytes (pH 2.5–4, 3.5–5, 5–7, 7–9, and 8–9.5) were from Fluka (Buchs, Switzerland), and tryptophol and *trans*, *trans-*farnesol was from Aldrich (St. Louis, Missouri). All other chemicals were of at least analytical grade.

### Red Blood Cell membrane preparation

Packed RBC were obtained from Canadian Blood Services, (Calgary, AB) and washed 3× with isotonic buffer (20 mM sodium phosphate pH 7.4, 0.9% NaCl). RBC ghosts were prepared according to the method of Chernomordik [29] with slight modifications. Cells were lysed osmotically in hypotonic lysis buffer (5 mM sodium phosphate pH 7.4, protease inhibitor cocktail [28], 5 mM DTT) for 20 minutes on ice. The lysate was flash frozen in a dry ice / ethanol bath, thawed, and membranes were collected by centrifugation (3000×g, 20 min, 4°C). Pellets were washed with wash buffer (20 mM sodium phosphate pH 8.5, protease inhibitor cocktail, 5 mM DTT) until supernatants were clear, and then subjected to a second round of hypotonic lysis and freeze-thaw. After washing until supernatants were clear, membranes were collected by centrifugation (3000×g, 40 min, 4°C), suspended in a minimal volume of wash buffer, and stored at -80°C. Before extraction, membrane isolates were washed with PBS containing protease inhibitors (PBS-PI) and pelleted (3 hours, 120 000×g, 4°C).

### Membrane preparations from mammalian tissues

Membranes were isolated as previously described [27]. Briefly, mouse brains or livers were flash frozen after dissection and stored at -80°C until needed. For the isolation of all cellular membranes, we applied a simple physical separation / fractionation protocol. Briefly, frozen tissues were thawed in hypotonic lysis buffer (20 mM HEPES pH 7.4, protease inhibitor cocktail, 10 mM sodium orthovanadate, 4 μM staurosporin, 4 μM cantharidin) and manually homogenized on ice with a polyethylene pestle in a 1.5 mL microcentrifuge tube. The homogenate was subjected to one round of freeze-thaw (-80°C), before being combined with an equal volume of 2×PBS to restore isotonicity. Membranes were collected by ultracentrifugation (3 hours, 120 000×g, 4°C), and were washed twice; pellets were resuspended in PBS-PI for each wash and collected by ultracentrifugation, as described above.

### Detergent Extractions

Detergent extraction buffers were prepared for 1D (7 M urea, 2 M thiourea, 9 mM Tris acetate pH 7.0, protease inhibitor cocktail, and detergent as indicated) or 2-DE (IEF buffer 1 containing 8 M urea, 2 M thiourea, protease inhibitor cocktail, and detergent as indicated [27]). Membrane pellets were resuspended by pipetting and vortexing. Extractions were incubated for 1 hour on ice, with periodic vortexing. Any insoluble material was separated by ultracentrifugation as previously described. Solubilized samples were assayed for total protein content using the EZQ Protein Quantitation Kit (Molecular Probes, Eugene, OR).

### Protein Quantification

Total protein was assayed using either the EZQ Protein Quantitation Kit or the RC DC Protein Assay Kit (BioRad, Hercules, CA). The RC DC assay was carried out according to manufacturers instructions in 96-well plates and absorbance was measured using the Wallac Victor2 Multilabel HTS Counter (PerkinElmer Life Sciences, Boston, MA). EZQ Protein Quantitation was carried out essentially according to manufacturers instructions except fluorescence was recorded by imaging on the Proexpress multiwavelength fluorescent imager (PerkinElmer, Boston MA) and spot fluorescence was quantified using ImageQuant 5.2 software (Molecular Dynamics, Sunnyvale, CA).

### 1D SDS-PAGE

1D SDS-PAGE was performed in mini gel format using the BioRad Protean II Electrophoresis system, essentially as described [30] with minor modifications [31]. Samples were normalized to 2 mg/ml in the appropriate extraction buffer, and then diluted 1:1 (v/v) with 2 × SDS sample buffer [30]. 10 μg total protein was loaded per well on 12.5%T separating gels with 5%T stacking gels, buffered with 375 mM Tris (pH 8.8) as described [31]. Gels were run at 125 V for 10 min to stack proteins, and then the voltage was reduced to 90 V to completion [32].

### 2-DE

Samples for IEF were normalized to 2 mg/ml with the appropriate IEF buffer, then combined 1:1 (v/v) with an ampholyte-containing IEF buffer (8 M urea, 2 M thiourea, 1% pH 3–10 broad range ampholytes, 0.2% each narrow range ampholytes (pH 2.5–4, 3.5–5, 5–7, 7–9, and 8–9.5) and detergent as indicated [27]), to introduce a working concentration of ampholytes to the sample.

Samples were sequentially reduced and alkylated essentially according to Herbert et al. [33,34] with some minor modifications. Briefly, the sample was reduced by the addition of TBP and DTT to final concentrations of 2.3 mM and 45 mM DTT, respectively, and incubated for 1 hour at 25°C. The reduced sample was then alkylated with 230 mM acrylamide monomer for 1 hour at 25°C. Immediately following alkylation, the sample was loaded onto IPG strips for passive hydration at 25°C (12 hours). IEF was carried out at 15°C using the BioRad Protean IEF Cell; voltage was ramped linearly to 4000 V (2 hours) and IEF was carried out at 4000 V (constant) for 37500 Vhours. After focusing, IPG strips were equilibrated essentially according to the manufacturer's instructions by sequential immersion in equilibration buffer (6 M urea, 2% SDS (w/v), 20 % glycerol (w/v), and 375 mM Tris pH 8.8) containing 130 mM DTT for 10 minutes, followed by equilibration buffer with 350 mM acrylamide monomer for 10 minutes. Following equilibration, IPG strips were loaded onto 12.5%T separating gels with 5%T stacking gels (buffered as described for 1D) and sealed in place with an agarose overlay (0.5% low melting agarose, 0.1% SDS and 375 mM Tris pH 8.8). SDS-PAGE was otherwise carried out as described for 1D SDS-PAGE.

### Image analysis

After electrophoresis, gels were fixed in 10% methanol, 7% acetic acid for 1 hour, washed thoroughly with water and stained with Sypro Ruby overnight. Gels were visualized using the Proexpress multiwavelength fluorescent imager (PerkinElmer, Boston MA). Quantitative image analysis was performed using Progenesis Workstation 2004 (Nonlinear Dynamics, Cambridge, UK). Parallel sets of gels were warped and matched by automated analysis, and volumes were normalized to a single spot consistent in size, shape, density and location across all gels.

## Abbreviations used

LPC (12:0 L-α-lysophosphatidylcholine), LPG (L-α-lysophosphatidylglycerol), LPE (L-α-lysophosphatidylethanolamine), LPS (L-α-lysophosphatidylserine), MEGA 8 (octanoyl-N-methylglucamide), MEGA 9 (nonanoyl-N-methylglucamide), MEGA 10 (decanoyl-N-methylglucamide), SB 3–10 (N-decyl-N,N-dimethyl-3-ammonio-1-propanesulfonate), ASB-14 (amidosulfobetaine-14), C12E8 (octaethylene glycol monododecyl ether), RBC (red blood cell)

## Competing interests

The author(s) declare that they have no competing interests.

## Authors' Contributions

MAC supervised the SDS-PAGE analysis, designed and carried out the 2-DE analyses, data acquisition and interpretation and prepared the manuscript. RHB contributed to the experimental design, sample preparation, 2-DE analyses, and participated in drafting the manuscript. JCL and KKH designed and carried out the SDS-PAGE analysis. JRC conceived and planned the study, and assisted in interpretation of data and final preparation of the manuscript. All authors read and approved the final manuscript.
